# Structural and biochemical insights of xylose MFS and SWEET transporters in microbial cell factories: challenges to lignocellulosic hydrolysates fermentation

**DOI:** 10.3389/fmicb.2024.1452240

**Published:** 2024-09-27

**Authors:** Iasmin Cartaxo Taveira, Cláudia Batista Carraro, Karoline Maria Vieira Nogueira, Lucas Matheus Soares Pereira, João Gabriel Ribeiro Bueno, Mateus Bernabe Fiamenghi, Leandro Vieira dos Santos, Roberto N. Silva

**Affiliations:** ^1^Molecular Biotechnology Laboratory, Department of Biochemistry and Immunology, Ribeirao Preto Medical School (FMRP), University of São Paulo, São Paulo, Brazil; ^2^Genetics and Molecular Biology Graduate Program, Institute of Biology, University of Campinas (UNICAMP), Campinas, Brazil; ^3^Manchester Institute of Biotechnology, University of Manchester, Manchester, United Kingdom

**Keywords:** xylose transporters, MFS, SWEET, protein engineering, second-generation ethanol, sugar uptake

## Abstract

The production of bioethanol from lignocellulosic biomass requires the efficient conversion of glucose and xylose to ethanol, a process that depends on the ability of microorganisms to internalize these sugars. Although glucose transporters exist in several species, xylose transporters are less common. Several types of transporters have been identified in diverse microorganisms, including members of the Major Facilitator Superfamily (MFS) and Sugars Will Eventually be Exported Transporter (SWEET) families. Considering that *Saccharomyces cerevisiae* lacks an effective xylose transport system, engineered yeast strains capable of efficiently consuming this sugar are critical for obtaining high ethanol yields. This article reviews the structure–function relationship of sugar transporters from the MFS and SWEET families. It provides information on several tools and approaches used to identify and characterize them to optimize xylose consumption and, consequently, second-generation ethanol production.

## 1 Introduction

Lignocellulose is a sustainable feedstock and renewable energy resource for the production of bioproducts and biofuels such as second-generation ethanol. It is a component of various agricultural residues such as sugarcane bagasse, rice straw, and corncobs, and offers great potential for use in ethanol biorefineries owing to its high availability and low cost. Therefore, the optimized use of lignocellulosic components can promote the transition to a bio-based economy (Moreno et al., 2022).

The economic viability of second-generation fuels depends on the ability of the yeast used in fermentation step, *Saccharomyces cerevisiae*, to assimilate substrates through saccharification. This species is widely used in industry because of its well-studied physiology and genome, exhibiting a high fermentation rate and high tolerance to fermentation stressors, such as variations in temperature, high ethanol concentrations, lower pH, and the presence of inhibitors released during biomass pretreatment. However, this yeast is unable to metabolize xylose naturally (Chu and Lee, 2007).

Thus, one of the main focuses of microbial engineering is to explore robust biological platforms capable of using different carbon sources such as glucose and xylose from cellulose and hemicellulose (Ko and Lee, 2018). Hemicellulose monomers, especially xylose, are abundant in plant biomass and are more easily obtained than glucose during saccharification because of the amorphous structure and low molecular weight of hemicellulose, which facilitates its hydrolysis (Jeffries, 1983; Haghighi Mood et al., 2013). The assimilation of xylose involves its conversion to xylulose and subsequent phosphorylation to xylulose-5-phosphate, which is metabolized via the pentose phosphate pathway. Some microorganisms such as *Aspergillus niger*, *Trichoderma reesei*, and *Pichia stipitis* can naturally metabolize xylose and convert it into ethanol.

One of the most commonly used approaches to optimize fermentation in *S. cerevisiae* is to engineer yeast strains with heterologous metabolic pathways that convert xylose to xylulose, such as xylose isomerase (XI) and xylose reductase/xylitol dehydrogenase (XR/XDH), which are present in organisms that inherently consume xylose (Silva et al., 2021; Cunha et al., 2019). However, the assimilation rate of xylose in recombinant *S. cerevisiae* was lower than that in yeasts that naturally use xylose, such as *P. stipitis* and *Candida shehatae*, mainly because of inefficient xylose transporters in the recombinant species (Gárdonyi et al., 2003).

The development of yeasts for efficient simultaneous consumption of glucose and xylose is essential for the bioethanol industry to increase its production. Xylose uptake in non-recombinant yeasts occurs via hexose transporters, and this sugar is usually consumed only after glucose is depleted from the medium due to the higher affinity of the hexose transporters for glucose (Patiño et al., 2019; Hou et al., 2017). In *S. cerevisiae*, for example, the glucose transporter with the highest affinity has a Km of ~1.5 mM (Hxt6 and Hxt7), whereas the xylose transporter with the highest affinity has a Km of ~130 mM (Hxt7). Indeed, *in silico* kinetic modeling analysis showed that the overexpression of xylose transporters is the most promising modification for enhancing ethanol production in recombinant yeasts (Hector et al., 2008). Therefore, several studies have focused on bioprospecting transporters from other species to increase the efficiency of D-xylose transport by industrial yeast strains (Parachin et al., 2011).

Many organisms have xylose transporters, such as plants, bacteria, filamentous fungi and yeasts. However, in this review we will focus on the study of transporters from organisms commonly used as cell factories and used in the context of bioethanol production. Filamentous fungi such as *T. reesei*, *A. niger*, *Aspergillus nidulans*, and *Neurospora crassa* can use complex polysaccharides because they can express and secrete a wide range of hydrolytic enzymes at high concentrations and possess various sugar transporters (STs) capable of transporting the sugar monomers produced during enzymatic hydrolysis into the cell (Hou et al., 2017; Jiang et al., 2020; Mattam et al., 2022). The two major classes of transporters in filamentous fungi are the ATP-Binding Cassette (ABC) superfamily and Major Facilitator Superfamily (MFS), which together account for half of the genes that encode transmembrane permeases (Nogueira et al., 2020). Recently, another family of STs, which had previously been identified in plants, animals, and bacteria, has also been identified in anaerobic fungi (*Neocallimastigomycota*). The Sugars Will Eventually be Exported Transporter (SWEET) superfamily has also been used to enhance xylose uptake in yeasts and enable the co-utilization of glucose and xylose (Jiang et al., 2019; Havukainen et al., 2021; Feng and Frommer, 2015).

Therefore, the expression of heterologous transporters in yeasts is one of the most promising approaches to increase xylose transport and bioethanol production, as noted in the literature. Different MFS and SWEET transporters have been successfully expressed in yeast, leading to increased D-xylose transport (Podolsky et al., 2022; Huang et al., 2015). Consequently, it is essential to thoroughly comprehend the structure and function of xylose transporters to understand how these proteins work and achieve the goal of highly efficient yeast in pentose transport.

Given this, this review will cover topics that consider the influence of the amino acid sequence on the function of xylose transporters. Furthermore, we will also discuss the influence of prospecting and engineering techniques for these proteins, compiling essential data for future work aimed at improving industrial strains of *S. cerevisiae*, as well as endogenous yeast transporters and those used for heterologous expression in the bioethanol context.

## 2 MFS and SWEET transporters: structural insights

**Figure 1:**
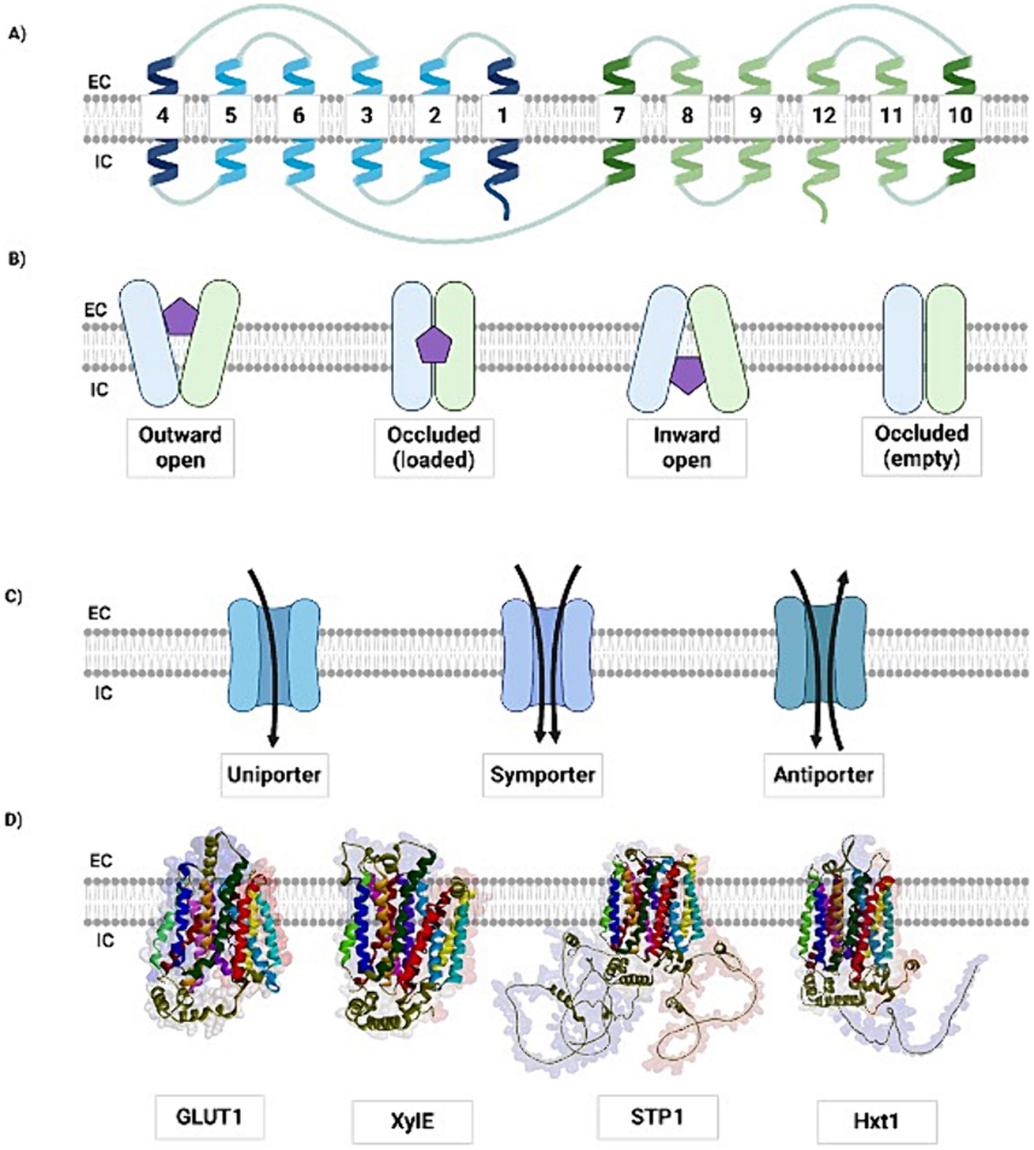
The MFS transporters are structurally and mechanistically conserved. **(A)** A schematic view of the canonical MFS topology showing the TMs from the N-terminal (blue) and C-terminal (green) domains. The TMs 1, 4, 7, and 10 (dark blue and dark green, respectively) are located in the central core of the transmembrane protein, being the ones that suffer the most part of conformational changes to interact, bind and release the substrate during its transportation across the membrane. **(B)** The different conformational states in which MFS transporters may occur to perform the general alternate access model. Here we represent the alternate access transport with the rocking-switch model, in which the MFS transporter suffers conformational changes to receive the extracellular sugar (purple pentagon), through its outward open conformation so that the protein can maintain the solute loaded in its occluded state and release it to the intracellular environment, in which the inward open state is presented. After this process, the protein may present itself empty in an occluded conformation, where it is ready to go through new conformational changes to receive new molecules to be transported. The N-and C-domains are represented in blue and green, respectively. **(C)** The different modes of solute transmembrane transporting. Most MFS are uniporters, in which the solute is transported in one direction one at a time, although there are symporters, capable of transporting the specific solute along with another substance, for instance, an ion; and also, antiporters, which carry two solutes at the same time, but in different directions. **(D)** Structural comparison among the MFS transporters from different species: the human GLUT1 (PDB ID: 4PYP), the bacterial XylE (PDB ID: 4GBZ), the yeast Hxt1 (modeled), and the filamentous fungus STP1 (modeled). Although there is low identity among the MFS transporters’ primary structures, the transmembrane arrangement is maintained across species, meanwhile, the outer and especially the cytoplasmic portions present less structural conservation. The models were predicted using AlphaFold. EC: extracellular; IC: intracellular. Created with BioRender (Supplementary material S1).

MFS transporters are essential for the efficient production of bioethanol via xylose fermentation. The highly ubiquitous and diverse MFS transporter family mainly comprises single-polypeptide secondary active transporters, with approximately 400–600 amino acids residues, typically arranged in 12 transmembrane helices, with variations containing 6, 14, or even 24 segments. The canonical topology (Figure 1A), which consists of 12 transmembrane helices (TMs), possesses segments organized into two domains comprising six TMs each, named the N-terminal and C-terminal domains. These two domains are connected by a flexible loop located in the cytosol, whereas the transmembrane segments, which are rich in hydrophobic amino acid residues, are mostly buried in the lipid bilayer, forming a channel that allows the substrate to cross the cell membrane. Each N-and C-terminal domain can be divided into two inverted repeats of three TMs that allow for different conformations: inward-open, outward-open, or occluded (Yan, 2015; Chen et al., 2015; Wang et al., 2020; Drew et al., 2021; Ferrada and Superti-Furga, 2022).

The different conformations are determined by the alternate access model, in which the protein structure undergoes conformational changes caused by substrate binding, alternating between the open and closed conformations, to transport the solute (Kaback and Smirnova, 2011; Drew and Boudker, 2016). Recent studies have attempted to explain how conformational alternation occurs (Jiang et al., 2019; Drew et al., 2021; Kaback and Smirnova, 2011; Drew and Boudker, 2016; Quistgaard et al., 2013; Västermark et al., 2015; Serdiuk et al., 2014; Sun et al., 2012). In the study of the mechanisms of the prototype bacterial transporter LacY, the authors proposed that LacY functions similarly to an enzyme, with the conceptual caution that possible conformers are referred to as the intermediate state, even though there is no transition state intermediate of the substrate itself (Serdiuk et al., 2014). While most TMs maintain a similar conformation both when the transmembrane transporter is present in an occluded conformation facing the cell interior or exterior, and when it is fully open in the same direction, the intra and extracellular helices bend to close the intra and extracellular portions of the protein, respectively (Madej, 2015). Occlusion is likely achieved because of the complete interaction between the substrate and the N-and C-terminal domains, since it may offer sufficient energy for changes in the transporter conformation (Serdiuk et al., 2014). To date, no experimentally validated structures of MFS transporters have been published as fully occluded. Therefore, the most accepted mechanism by which MFS proteins mediate solute transport across the membrane is based on the alternate access model (Figure 1B) (Drew and Boudker, 2016; Bartels et al., 2021). Furthermore, despite different transport models, MFS transporters can function as uniporters, symporters, or antiporters, as illustrated in Figure 1C (Yan, 2015; Chen et al., 2015).

Although there are diverse mechanisms by which MFS transporters perform their functions, their transmembrane structural arrangement is highly conserved (Figure 1D) (Bartels et al., 2021; Zhang X. C. et al., 2015). Topological conservation provides a foundation for understanding the molecular mechanisms underlying substrate transport through these transporters (Wang et al., 2020). Vishwakarma et al. (2018) verified that most of the conservation among MFS transporters is related to TMs arrangement, whereas the cytoplasmic and extracellular regions present higher degrees of variation, probably because of evolutionary divergence among species. However, comparison of these protein sequences is challenging because of poor primary sequence conservation, mostly presenting identity values below 25%. Despite the differences in amino acid sequences and lateral chains, functional conservation was verified regarding sugar-binding sites among the different MFS transporters, regardless of their diverse functions and transported solutes (Vishwakarma et al., 2018).

The conservation of binding sites is important for the identification of functional residues in the protein structure, although they may differ in the primary sequence. Furthermore, the presence of highly conserved residues, especially in transmembrane regions, indicates the need for structural maintenance and folding, and consequently, functional preservation of the protein. For example, TMs 1, 4, 7, and 10 are at the center of all MFS transporters and are directly involved in the transport of carbohydrates, with most of their residues identified as interactors with the substrate (Drew and Boudker, 2016; Yan, 2013). Similarly, the conservation of charged residues might be related to the transport of ions through the protein, whereas proline-or glycine-rich regions, for instance, may represent higher degrees of conformational changes by introducing more flexibility to the biomolecule and enabling substrate accommodation, thus working as substrate-gating regions and obstructing its externalization (Bartels et al., 2021; Vishwakarma et al., 2018). Moreover, TMs 2, 5, 8, and 11 serve as bridges between the N-and C-domains and participate in substrate binding and transport, while TMs 3, 6, 9, and 12 provide structural stability to the protein (Yan, 2015; Yan, 2013; Reider Apel et al., 2016; Young et al., 2014).

Considering the functional and topological conservation of MFS transporters, an example of this feature is the homology between the human GLUT1 intracellular helical domain and bacterial XylE, which may facilitate the closing of the intracellular gate in the outwardly closed conformation. Although XylE contains an intracellular domain with four helices, in both species, the hexose is bound between the protein N-and C-termini, and this interaction is organized mainly by polar and aromatic residues at the C-terminal domain. Considering this, it has been proposed that the sugar is recognized at the C-terminus, and that the N-terminus undergoes conformational changes to present the substrate to the alternate side of the membrane (Chen et al., 2015; Sun et al., 2012; Madej, 2015). Interestingly, Jiang et al. (2019) took advantage of the conserved feature of the MFS and used XylE as a tool for studying the mechanisms relying on glucose transport through GLUTs by engineering the former to constitutively exhibit specific conformations to enable the evaluation of the binding affinities for the substrates (Jiang et al., 2019).

XylE is an H+/xylose symporter protein found in some bacteria such as *Escherichia coli*. The structure of XylE has already been characterized in different conformations, and its engineering has been studied to increase xylose consumption using various techniques such as directed evolution and computational and experimental methods (Jiang et al., 2019; Sun et al., 2012; Henderson et al., 2021; de Valk et al., 2022; Wisedchaisri et al., 2014). Since the elucidation of its structure, most studies aimed at understanding the transport mechanisms of xylose and glucose have focused on the interactions of this protein with sugars (Sun et al., 2012; Wisedchaisri et al., 2014). Wisedchaisri et al. (2014) noticed that the shift between the inward and outward open conformations is comprised of an asymmetrical movement that allows the substrate to bind to a specific site or be released from it; they proposed that the proton-coupled mechanism of substrate internalization involves proton transfer from specific aspartate residues to water, so it can be released into the cytoplasm before the sugar release itself (Wisedchaisri et al., 2014). These inferences are consistent with those reported for the proton-coupled mechanism of the MFS transporter Mal11 in *S. cerevisiae* in other studies (Henderson et al., 2021; de Valk et al., 2022).

SWEET proteins are a family of membrane transporter proteins found in plants, animals, bacteria, and fungi that are responsible for the transport of sugars across the cell membrane. Although SWEET transporters are mainly responsible for the efflux of solutes in plants, they are also involved in the uptake of various sugars, including monosaccharides, such as glucose and fructose, and disaccharides, such as maltose and sucrose, into fungal cells. They play a crucial role in fungal nutrition and are important in the biotechnological industry because they can be utilized to produce biofuels and other value-added products from plant-derived sugars. SWEET transporters can be categorized as members of the MFS superfamily of the MtN3/saliva family. Members of the SWEET family can be classified according to their sugar preference into four clades: clades I and II consist of hexose transporters, clade III consists mainly of sucrose transporters, and clade IV consists of fructose-preferring transporters (Feng and Frommer, 2015; Chen et al., 2015).

**Figure 2:**
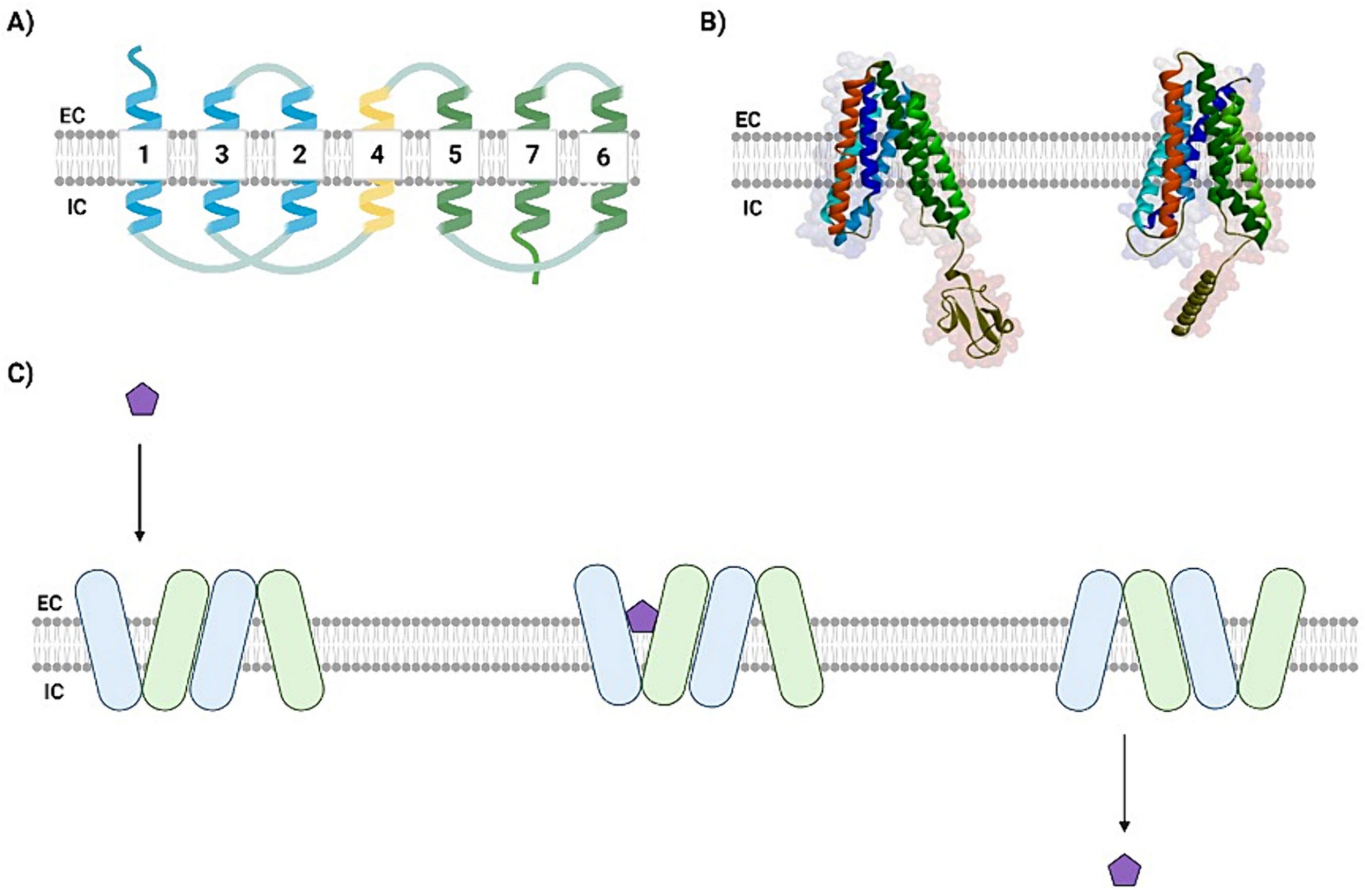
SWEETs present a well-conserved transmembrane domain arrangement. **(A)** A schematic view of SWEETs topology showing the six TMs separated by a less conserved intra-TM domain (yellow). The N-terminal domain is colored blue, and the C-terminal domain is colored in green. **(B)** Tridimensional comparison between the crystallographic image of AtSWEET13 (PDB ID: 5XPD) and the Neocallimastigomycota NcSWEET1 (modeled). The N-terminal domain comprises the blue helices, while the C-terminal is represented by the green ones, both portions separated by the intra-TM domain (in red). The NcSWEET1 model was predicted using AlphaPhold. **(C)** The revolving-door mechanism, proposed by Han et al. (2017). The sugar (purple pentagon) binds to the binding cavity in the first SWEET monomer transmembrane domain, inducing the closing of its cytosolic gate and the conformational change of a second SWEET monomer, so that while the sugar is released from the first monomer, the next is prepared to receive a new molecule and start a new transport cycle. EC: extracellular; IC: intracellular. Created with BioRender (Supplementary material S2).

Structurally, eukaryotic SWEETs comprise a direct repeat of three TMs separated by a single and less conserved TM domain (Figures 2A,B), whereas in bacteria (semi-SWEETs), these transporters are much smaller in terms of amino acid length, and the formation of a dimer is the minimal condition that causes the translocation of pores in semi-SWEETs (Xu et al., 2014). SWEETs contain an extracellular N-terminal domain and an intracellular C-terminus with positively charged residues that are more prominent in the cytosolic loop (Ji et al., 2022). As with MFS transporters, SWEETs generally present low primary sequence identity, despite their conserved function (Jia et al., 2018). Some authors have suggested that the topological differences between MFS and SWEETs are crucial for the ability of the latter to import and export sugars into the extracellular environment (Ji et al., 2022). However, notwithstanding the differences between the sequences of the N-and C-domains, both families present double pseudosymmetry perpendicular to the membrane plane. Furthermore, SWEET proteins possess a highly phosphorylated C-terminal end that may interact with other proteins, suggesting that SWEETs proteins may also act as transceptors (Chen et al., 2015).

In contrast to MFS transporters, additional energy is not required for sugars to be transported across SWEETs because transport is based on the concentration gradient, mainly via uniporters (Bartels et al., 2021). However, similar to the MFS transporters, the resolution of SWEET crystal structures suggests that these transporters undergo a rigid-body rocking-type movement while transporting sugars, as the presence of different open and occluded conformations has been verified (Lee et al., 2015). Han et al. (2017) performed smFRET analyses and concluded that a cooperative “revolving door” mechanism may promote conformational changes in both monomers that allow the sugar to be carried to the opposite side (Figure 2C) (Han et al., 2017). Considering the possibility of transporting both mono and disaccharides, it is interesting to point out that larger cavities in the transporter can allow both mono and disaccharides to fit in, and the opposite is true, although the fitting of the monosaccharide in the specific larger cavity may not indicate better transport, as there may not be optimal interactions between the smaller sugar and cavity residues (Chen et al., 2015).

Taken together, it is possible to confirm that knowledge of the structural features of transmembrane transporters is essential for understanding the mechanisms by which sugars can cross the cell membrane, making it possible to engineer these proteins to shift carbohydrate transport so that the most advantageous sugars are preferred over others. Concerning bioethanol production, a deep knowledge of ST structural biology provides the possibility of optimizing xylose uptake, which is highly beneficial for fermentative processes.

## 3 Motifs and conserved amino acids involved in xylose transport

Young et al. (2012) reported that hydrophobic, nonpolar, and moderate-to-large-sized amino acids favor xylose selectivity because pentose is smaller than glucose (Young et al., 2012). Membrane proteins are typically well-conserved and have similar properties, such as folding and addressing information (Li et al., 2014). The MFS family is the most studied sugar transporter family and is heterologously expressed in *S. cerevisiae*. However, studies on SWEET transporters have gained attention, and the proteins of this family present a topology of triple helix bundles comparable to the helices of the MFS family, although they are architecturally different (Xu et al., 2014; Jeckelmann and Erni, 2020). According to Xu et al. (2014), this could be because the SWEET and MFS transporters, for example, evolved from a single ancestral protein with this triple helix bundle, or that this represents a convergent evolution (Xu et al., 2014).

**Figure 3:**
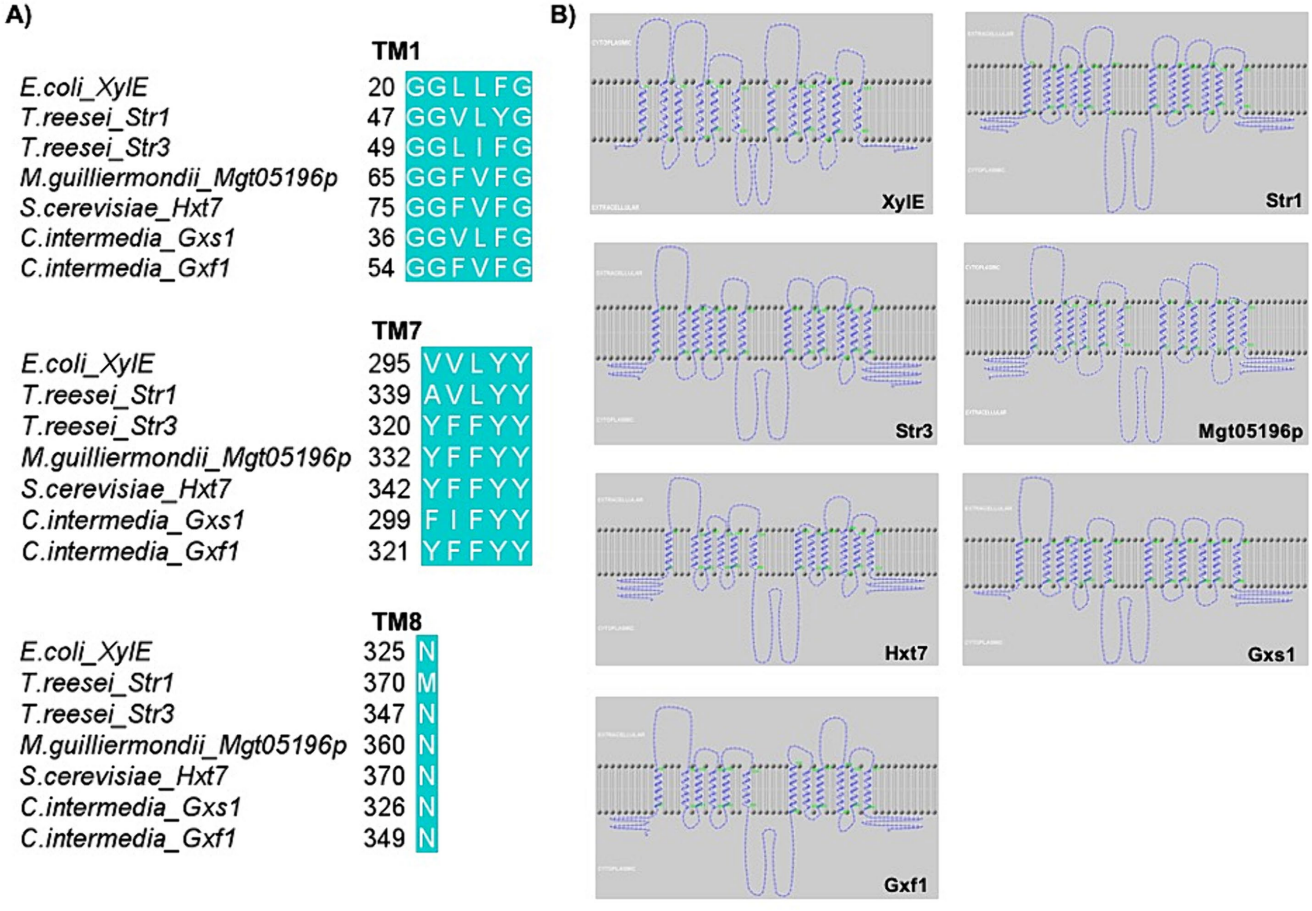
MFS are proteins with conserved domains and amino acids, which is reflected in functional similarities. **(A)** Alignment of the amino acid sequences of XylE, Str1, Str3, Mgt05196p, Hxt7, Gxs1, and Gxf1 using the software BioEdit by ClustalW method and visualized in Jalview 2.11.2.6 software (Waterhouse et al., 2009). **(B)** Topology of the XylE, Str1, Str3, Mgt05196p, Hxt7, Gxs1, and Gxf1 chains. The prediction of the topology and transmembrane helices was done in the HMMtop webserver (Tusnády and Simon, 1998; Tusnády and Simon, 2001) and the image was generated in TMRPres2D (Spyropoulos et al., 2004).

Therefore, several studies have investigated if amino acids and motifs that may be important for xylose transport are conserved. These studies generally cover topics ranging from the conservation of amino acids directly related to the translocation of sugars to the conservation of amino acids related to the effectiveness of proteins (Reider Apel et al., 2016; Young et al., 2014). The most explored motifs and amino acids for substitutions and edits were GG/FXXXG (described in *C. intermedia* Gxs1) (Lee et al., 2015), YFFYY (described in Mgt05196p from *Meyerozyma guilliermondi*) (Wang C. et al., 2015); and N370 in Hxt7 from *S. cerevisiae* (Figure 3) (Farwick et al., 2014), as can be seen in Table 1. As an example, Figure 3A shows the conservation of these motifs and amino acids in different species, such as filamentous fungi (Str1 and Str3 from *T. reesei*), yeasts (Gxf1 and Gxs1 from *C. intermedia*; Mgt05196p from *M. guilliermondi*), and bacteria (XylE from *E. coli*). Furthermore, the representation of these protein topologies (Figure 3B) revealed the importance of this conservation in TMs 1, 7, and 8, which are important for substrate binding.

**Table 1:** Xylose transporters and their characteristics.

Transporter	Organism	Motif or amino acid mutation	Kinetics values (xylose)	Phenotype	Observations	Engineering methods	Ref.
Gxs1	*Candida intermedia*	G36G37V38L39F40G41	Km = 0.0256 ± 0.0659 mM; Vmáx = 7.23 ± 0.6 nmol min-1 gDCW-1.	Motif involved with substrate selectivity.		Wild type motif	Young et al. (2014)
		G36G37F38I39M40G41	Km = 0.721 ± 0.116 mM; Vmáx = 15.01 ± 2.38 nmol min-1 gDCW-1.	Reduction in xylose affinity, compensated by an increase in transport capacity. Guarantees greater efficiency in the simultaneous consumption of glucose and xylose.	Original motif: G36G37V38L39F40G41	Site-directed mutagenesis	Young et al. (2014)
		F40M	Km = 0.721 ± 0.116 mM; Vmáx = 15.01 ± 2.38 nmol min-1 gDCW-1.	Prevents yeast growth on all carbon sources except xylose and galactose.	Original motif: G36G37V38L39F40G41	Site-directed mutagenesis	Young et al. (2014)
		F229I230F231Y232Y233	Not measured.	Motif rich in aromatic amino acid residues and its presence is necessary to ensure xylose selectivity.	It differs from the YFFYY motif initially described in Mgt05196p.	Wild type motif	This work
		E438K	Km = 1.25 ± 0.32 mM; Vmáx = 10.91 ± 1.44 nmol min-1 gDCW-1.	Increased xylose growth rate as well as improved transport affinity and efficiency.	Authors do not discuss the mutation much and attribute it to a similar profile to the E538K mutation in Xut3.	PCR error prone	Young et al. (2012)
Mgt05196p	*Meyerozyma guilliermondi*	Y332F333F334Y335Y336	Not measured.	Motif rich in aromatic amino acid residues and its presence is necessary to ensure efficency of growth in xylose.	First description of the YFFYY motif as important for xylose stereoselectivity. Located in TM7.	Wild type motif	This work
		Replacement of any of Y332F333F334Y335Y336 with alanine (A)	Not measured.	Significantly reduces the ability to transport xylose.	The tyrosine residues near the end of the motif (Y335Y336) are highly conserved. Substitution of these residues leads to poorer growth on xylose.	Site-directed mutagenesis	Wang C. et al. (2015)
		R164A	Not measured.	Inactivates transport activity, this profile also observed in XylE.	In XylE R133A substitution is observed to disable the construction of a salt bridge and is involved in the protonation of XylE in *E. coli* to control the outward-facing or inward-facing open conformation.	Site-directed mutagenesis	Wang C. et al. (2015)
Hxt7	*Saccharomyces cerevisiae*	N370	Km = 200.3 ± 13.2 mM; Vmáx = 67.0 ± 2.0 nmol·min − 1·mg − 1.	Crucial for glucose binding and modifications at this site can have a significant impact on the transporter’s ability to transport xylose.	Located on the extracellular side of the central binding pocket in TM8. Mutations in regions adjacent to the binding sites may affect the co-consumption of glucose and xylose.	Wild type residue	Farwick et al. (2014)
		N370A	Not measured.	Growth on xylose with high affinity; drastic reduction of inhibition by glucose.		ALE, Site-directed mutagenesis	Farwick et al. (2014)
		N370S	Km = 169.9 ± 26.3 mM; Vmáx = 24.1 ± 1.6 nmol·min − 1·mg − 1.	Increases xylose affinity and decreases glucose affinity, similar to the N376V mutation in Gal2.		ALE, Site-directed mutagenesis	Farwick et al. (2014)
		F79S	Km = 228.8 ± 45.9 mM; Vmáx = 186.4 ± 20.1 nmol•min − 1•mg − 1.	Reduction in xylose affinity, compensated by an increase in transport capacity. Guarantees greater efficiency in the simultaneous consumption of glucose and xylose.	The residue affects the transport biding with xylose.	Evolutionary engineering	Reider Apel et al. (2016)
XylE	*Eschericia coli*	F24	Km = 0.47 ± 0.05 mM.	Direct biding with xylose.	Original motif: G20G21L22L23F24G25	Wild type residue	Sun et al. (2012)
		T219	Km = 0.47 ± 0.05 mM.	Important for glucose binding, being potential targets for improving xylose transport.	Located on the extracellular side of the central binding pocket in TM 5. Mutations in regions adjacent to the binding sites may affect the co-consumption of glucose and xylose.	Wild type residue	Farwick et al. (2014)
Gxf1	*Candia intermedia*	YFFYY	Not measured.	Motif rich in aromatic amino acid residues and its presence is necessary to ensure efficency of growth in xylose.	Located in TM7.	Wild type motif	This work
Hxt1	*Ogataea polymorpha*	N358A	Not measured.	Together with the replacement of N-terminal lysine residues by arginine residues, it resulted in the simultaneous utilization of xylose and glucose during the co-fermentation of these sugars by O. polymorpha.	Endogenous transporter of O. polymorpha.	Overexpression of endogenous transporter.	Vasylyshyn et al. (2020)
Gal2	*Saccharomyces cerevisiae*	N376	Km = 225.6 ± 15.8 mM; Vmáx = 91.3 ± 3.2 nmol·min − 1·mg − 1.	Crucial for glucose binding and modifications at this site can have a significant impact on the transporter’s ability to transport xylose.	Located on the extracellular side of the central binding pocket in TM8. Mutations in regions adjacent to the binding sites may affect the co-consumption of glucose and xylose.	Wild type residue	Farwick et al. (2014)
		N376V	Km = 91.4 ± 8.9 mM; Vmáx = 37.3 ± 1.3 nmol·min − 1·mg − 1.	Increased affinity for xylose and decreased affinity for glucose.		Site-directed mutagenesis	Farwick et al. (2014)
		N376F	Km = 168.3 ± 31.6 mM; Vmáx = 28.4 ± 2.3 nmol·min − 1·mg − 1.	It eliminates the inhibition of xylose transport by glucose, indicating that the inhibition mechanism involves steric exclusion and competitive inhibition.		Site-directed mutagenesis	Farwick et al. (2014)
		T219	Km = 225.6 ± 15.8 mM; Vmáx = 91.3 ± 3.2 nmol·min − 1·mg − 1.	Important for glucose binding, being potential targets for improving xylose transport.	Located on the extracellular side of the central binding pocket in TM 5. Mutations in regions adjacent to the binding sites may affect the co-consumption of glucose and xylose.	Wild type residue	Farwick et al. (2014)
		M435I	Not measured.	Improves the growth of yeast modified in arabinose, which is a pentose like xylose.	It gives greater stereoselectivity to the transporter.	Site-directed mutagenesis	Wang et al. (2016)
		L311R, L301R, K310R, N314D, M435T, T386A	Not measured.	Set of epistatic mutations crucial for improving the Gal2 phenotype.	Localized in loop regions or transmembrane segments (TMs) of the Gal2 protein crucial for substrate recognition and transport.	PCR error prone	Reznicek et al. (2015)
		L311R	Not measured.	Result in faster growth on xylose compared to wild-type Gal2.	Located in the loop region between TM6/7.	PCR error prone	Reznicek et al. (2015)
		L301R, K310R, N314D, M435T	Not measured.	These mutations, together with the mutations present in variant 1.1, were identified in variant 2.1. These mutations led to increased xylose uptake and decreased glucose specificity. Variant 2.1 exhibited the most significant growth at low xylose concentrations and enhanced sugar metabolism of glucose and xylose simultaneously.	The first three mutations are located in the large loop 6/7, while M435T is at the interface with loop 9/10.	PCR error prone	Reznicek et al. (2015)
		T386A	Not measured.	Allows growth on xylose without inhibiting glucose.	Located on the extracellular side of the central binding pocket in TM8. Mutations in regions adjacent to the binding sites may affect the co-consumption of glucose and xylose.	PCR error prone	Reznicek et al. (2015)
		Y446	Km = 320.5 mM; Vmáx = 88.72 nmol/min*mg.	Responsible for the formation of hydrogen bonds with xylose and glucose in Gal2. The formation of this polar bond favors glucose transport and decreases the interaction of xylose with the transporter’s N and C terminal domains. Therefore, it is the target of mutations that favor xylose transport.	Located on the extracellular side of the central binding pocket in TM11. Mutations in regions adjacent to the binding sites may affect the co-consumption of glucose and xylose.	Wild type residue	Kuanyshev et al. (2021)
Hxt36	*Synthetic*	N367	Km = 107.9 ± 12.1 mM; Vmáx = 62.5 ± 5.9 nmol/mg DW.min.	Crucial for glucose binding and influences the ability to co-consume glucose and xylose.	Located on the extracellular side of the central binding pocket in TM8. Mutations in regions adjacent to the binding sites may affect the co-consumption of glucose and xylose.	Wild type residue	Nijland et al. (2014)
		N367A	Km = 39.8 ± 5.6 mM; Vmáx = 23.0 ± 3.0 nmol/mg DW.min.	This mutation ensures growth on xylose with high affinity, drastically reducing inhibition by glucose.		ALE, Site-directed mutagenesis	Nijland et al. (2014)
		N367I	Km = 24.9 ± 3.4 mM; Vmáx = 29.1 ± 0.4 nmol/mg DW.min.	This mutation increases the selectivity for xylose, preventing glucose from binding to its site, without affecting xylose binding.		ALE, Site-directed mutagenesis	Nijland et al. (2014)
Hxt2	*Saccharomyces cerevisiae*	T339P/M420I	Not measured.	Together the mutationS in Hxt2 increases yeast growth in the presence of xylose.	Important for xylose specificity because proline is cyclic and nonpolar. Located in TM7.	Saturation mutagenesis	Wang et al. (2016)
Xut3	*Scheffersomyces stipitis*	E538K	Km = 2.02 ± 0.40 mM; Vmáx = 15.67 ± 0.87 nmol/min/gmDCW.	Increased xylose growth rate as well as improved transport affinity and efficiency.	Amino acid residue is located in the N-terminal cytosolic loop structure and appears to be responsible for the large changes in transport phenotypes observed. Its location indicates that it enhances the release of xylose into the intracellular medium or perhaps alters the overall conformation of the protein.	PCR error prone	Young et al. (2012)
Ncsweet 1	*Neocallimastix californiae*	P52A	Not measured.	Loss of transporter function.	Fundamental for conformational changes in transport.	Site-directed mutagenesis	Podolsky et al. (2021)
		N201A	Not measured	Loss of transporter function.	Fundamental for conformational changes in transport.	Site-directed mutagenesis	Podolsky et al. (2021)
		P154A	Not measured.	Reduction of transporter function.	Fundamental for conformational changes in transport.	Site-directed mutagenesis	Podolsky et al. (2021)
		W185G	Not measured.	Reduction of transporter function.	Fundamental for conformational changes in transport.	Site-directed mutagenesis	Podolsky et al. (2021)
LST1_205437	*Lipomyces starkeyi*	F433	Km = 145.3 mM; Vmáx = 76.80.	Allows co-consumption of glucose and xylose.	Located at the position equivalent to amino acid Y446 in the Gal2 transporter, F433 stands out for being an apolar amino acid that does not form any polar bond with the substrates, favoring the transport of xylose in LST1_205437.	Wild type residue	Kuanyshev et al. (2021)

The first TM helix, which is a part of the MFS transporter structure, contains the GG/FXXXG motif, which has received most attention. The presence of aromatic residues, such as phenylalanine, is particularly notable because it is closely related to xylose selectivity. Thus, when analyzing the alignment of wild-type transporters, the occurrence of this amino acid at the final position of the weakly conserved amino acids (GGXXFG) is noteworthy (Table 1) (Young et al., 2014; Bueno et al., 2020; Sloothaak et al., 2016). The equivalent residue in XylE, F24, which is part of the transporter binding pocket, causes significant changes in transporter function when mutated. However, Young et al. (2014) found that the F40M mutation in Gxs1 (wild-type motif: G36 G37 V38 L39 F40 G41) prevented yeast expressing the transporter from growing in all carbon sources except xylose and galactose (Young et al., 2014). Furthermore, when rewriting the motif G36 G37 V38 L39 F40 G41 to G36 G37 F38 I39 M40 G41, a reduction in affinity for xylose was observed, which was compensated by increased transport capacity. Thus, this set of alterations ensured that the mutant transporter was most efficient in co-consuming glucose and xylose. Similarly, substituting this residue in Hxt7 (F79S) altered the kinetics of xylose transport (Reider Apel et al., 2016). Hence, the importance of uncharged amino acids is evident, and specific sets of changes at amino acid positions favor xylose transport.

Initially discovered in the transporter Mgt05196p, the need for the aromatic residue-rich motif Y332F333F334Y335Y336 to ensure selectivity for xylose was demonstrated by the fact that altering these residues to alanine significantly lowered the ability of this transporter to uptake xylose (Table 1) (Wang C. et al., 2015). However, the alignments showed that the tyrosine residues near the terminus of the motif were highly conserved, and the other amino acids in the motif exhibited substantial variability (Sloothaak et al., 2016). Although Gxf1 is regarded as a model for xylose transport and contains the reported conserved motif, it has a higher Km_xylose_ than Gxs1 (48.7 + −6.5 mM and 0.012 + −0.004 mM, respectively) (Leandro et al., 2006). Gxs1 possesses F229I230F231Y232Y233 instead of the previously described motif. As a result, it is reasonable to conclude that the two tyrosine residues at the initial position of the motif are crucial for transporter operation and that the inclusion of larger neutral amino acids at the remaining locations would be sufficient.

In addition to the amino acid motifs mentioned above, point mutations can have a significant impact on xylose transport (Table 1) (Wang C. et al., 2015; Bueno et al., 2020). The engineering of STs in the yeast *Ogataea polymorpha* by site-directed mutagenesis of N358A, along with the substitution of N-terminal lysine residues in its Hxt1 homolog, improved the utilization of xylose, in contrast to the wild-type (Vasylyshyn et al., 2020). Similarly, Lian et al. (2018) discussed the tools used in Hxt1 de *S. cerevisiae* protein engineering to improve xylose uptake and fermentation in yeast (Lian et al., 2018). Farwick et al. (2014) showed that residues directly involved in the binding site of glucose and xylose, such as N376, are highly sensitive to modifications in Gal2, sometimes compromising transporter function, although amino acid modifications in the adjacent regions of the binding sites may affect the co-consumption of glucose and xylose (Farwick et al., 2014). Meanwhile, the application of random mutations in specific residues in Gal2, such as T386, may cause mutants to exhibit higher growth rates at low xylose concentrations, making it possible to simultaneously consume xylose along with glucose (Nijland and Driessen, 2020; Reznicek et al., 2015). Moreover, the substitution of N370S in Hxt7 increased its affinity for xylose and decreased its affinity for glucose, which was similar to that observed for the N376V mutation in Gal2, whereas the N376F substitution completely abolished the inhibition by glucose (Wang C. et al., 2015; Farwick et al., 2014). The preservation of the asparagine residue in this location rather than the presence of a nonpolar or uncharged amino acid is therefore justifiable, given the importance of glucose in the energy metabolism of almost all living organisms. Nevertheless, this residue is regarded as an interesting engineering target, because the substitution of this amino acid is functional.

Similarly, a highly conserved asparagine residue in *S. cerevisiae* was recently identified as essential for the maintenance of Hxt36 and Gal2 affinities for glucose. Both Hxt36 and Gal2 have three-dimensional structures modeled based on the XylE template bound to xylose. Interestingly, both modeled structures present a glucose-binding site close to residues N367 in Hxt36 and N376 in Gal2, in which mutations to more hydrophobic or sterically clashing amino acids in this region can influence the binding of this sugar, although the affinity for xylose may remain the same (Farwick et al., 2014; Nijland and Driessen, 2020). Nijland et al. (2014) demonstrated the importance of N367 in the Hxt36 chimeric transporter for xylose transport, although its degree of conservation is evident (Reider Apel et al., 2016; Wang C. et al., 2015; Farwick et al., 2014; Bueno et al., 2020; Nijland et al., 2014). Since the asparagine residue at this location is crucial for glucose binding to transporters, several researchers have focused on replacing it with amino acids that boost xylose selectivity, which seems to be related to amino acid polarity and size (Nijland et al., 2014). Concurrently, its replacement with alanine ensures growth on xylose with high affinity, drastically reducing inhibition by glucose (Sun et al., 2012; Nijland et al., 2014).

A recently characterized hexose transporter, Cs4130 from *Candida sojae*, possesses a highly conserved sugar-binding region compared to Gxf1 and XylE, and alterations in binding affinity suggest that the amino acid residues adjacent to the binding site may interfere with protein-sugar interactions. Bueno et al. (2020) compared the binding affinities from docking experiments involving Cs4130 to the available data for XylE and found important residues and motifs related to the selectivity for xylose, allowing the manipulation of xylose consumption as the sole carbon source or along with glucose, as discussed below.

Other amino acids have been identified as key xylose transporters, although these have been little explored, such as T219 in XylE and Gal2, such as T213 in Hxt7. These amino acid residues are located on the extracellular side of the central binding pocket in TM 5. They are known to be important because of their contribution to glucose binding, serving as probable targets for the improvement of xylose transport (Farwick et al., 2014; Kasahara et al., 2004). In contrast, residue R164 in Mgt05196p from *Meyerozyma guilliermondi* (R133 in XylE) is a conserved amino acid involved in the formation of a salt bridge that controls the position of the protein. Changes in this residue position for a nonpolar amino acid disabled the transport activity, with the same profile observed in XylE (Sun et al., 2012; Wang C. et al., 2015; Sloothaak et al., 2016). Wang C. et al. (2015) showed that this residue is not involved in the coupled H+ symport in Mgt05196p, which was corroborated by the presence of this conserved amino acid in Gxf1 (a facilitator) in alignment analyses.

Additionally, the amino acids T339 and M420 in Hxt2 (*S. cerevisiae*) are important for xylose specificity since the substitutions T339P and M420I in a mutant protein with both mutations could increase yeast growth in the presence of xylose, while decreasing it in Hxt-null *S. cerevisiae*. T339 is positioned at TM7, and M420 is positioned at TM10, both of which are known to be directly involved in substrate binding in MFS and are highly conserved in xylose transporters (Wang et al., 2016; Tamayo Rojas et al., 2021). In addition, the mutation M435I in Gal2 (T339P in Hxt2) showed the same profile as that observed in Hxt2, conferring improved growth of the engineered yeast in arabinose, probably because isoleucine is an amino acid with a larger side chain, providing more stereoselectivity. Thus, it is possible to infer the importance of the conserved amino acid M420 in pentose stereoselectivity (Wang et al., 2016).

Furthermore, the study of motifs and amino acids and their conformations is crucial for understanding the mechanisms of transporter selectivity for different sugars. Kuanyshev et al. (2021) used the GG/FXXXG motif and N370 amino acid of Hxt7 to search for glucose and xylose transporters in oleaginous yeasts and identified some transporters belonging to the SWEET family. The analysis of these MFS transporter motifs found in SWEETs may also be understood as a compelling rational method for prospecting xylose transporters if the transporters of both families share a common ancestor and functional qualities.

## 4 Co-consumption of glucose and xylose

MFS transporters can recognize and transport more than one type of substrate across the cell membrane. For instance, the STP1 transporter from *T. reesei* accepts glucose and cellobiose, whereas the XtrD transporter from *A. nidulans* transports xylose, glucose, galactose, and mannose (Zhang W. et al., 2015). Considering xylose fermentation, these transporters play a key role in the uptake of xylose to generate bioethanol. Because of the limited ability of some microorganisms to transport xylose, the engineering of MFS transporters using techniques such as overexpression, site-directed mutagenesis, or co-expression of multiple hexose transporters has been continuously investigated to optimize the usage of this sugar to improve the feasibility of biofuel production (Table 1) (Reider Apel et al., 2016; Young et al., 2012; van der Hoek and Borodina, 2020; Sharma et al., 2018; Kricka et al., 2014; dos Reis et al., 2016; Oh and Jin, 2020; Colabardini et al., 2014).

The co-consumption of glucose and xylose is essential to guarantee high fermentation rates of lignocellulosic biomass, precisely because transport is the first step toward metabolism. However, *S. cerevisiae* can neither naturally metabolize xylose nor possess specific transporters for this sugar (Kuyper et al., 2005; Saloheimo et al., 2007; Papapetridis et al., 2018). Hence, xylose is transported through endogenous yeast transporters such as Hxt-1, 2, 4, 5, 7, and Gal2, where Hxt7 is the endogenous transporter with the lowest Km value for xylose (Reider Apel et al., 2016; Saloheimo et al., 2007). In general, these hexose transporters have low affinity for xylose (Km:880 and 130 mM), but some of them exhibit high internalization rates (Vmax:750 and 110 nmol/min/mg-protein) (Table 1) (Saloheimo et al., 2007; Tanino et al., 2012).

Because of the inefficiency of xylose transporting systems in yeast, many studies have investigated more specific transporters for this pentose to be heterologously expressed in *S. cerevisiae*. Some studies have focused on identifying organisms that can naturally metabolize xylose, such as the filamentous fungi *Aspergillus* spp. (Sloothaak et al., 2016; Colabardini et al., 2014), *T. reesei* (Huang et al., 2015), *N. crassa* (Li et al., 2014), in addition to the yeasts *Scheffersomyces stipitis* (Weierstall et al., 1999; Moon et al., 2013), and *Candida intermedia* (Leandro et al., 2006). However, most wild-type xylose transporters have a higher affinity for glucose than for pentoses (Young et al., 2012). Many studies have aimed to increase the selectivity of these transporters for xylose in addition to reducing transport inhibition owing to competition with glucose through various strategies for protein engineering (Qiao et al., 2021), as discussed in the next section of this review.

Considering the evolutionary aspect, it is natural to assume that genes that encode STs capable of transporting glucose with higher efficiency may be preserved. Thus, both the transport and metabolism of sugars, such as xylose, are inhibited by glucose (Zhao et al., 2020). Many studies have demonstrated the diauxic growth pattern of *S. cerevisiae* when cultivated in mixtures of glucose and other sugars, with glucose being consumed first (Kuyper et al., 2005; Lane et al., 2018; Peng et al., 2015). In this context, even transporters from organisms that can naturally metabolize xylose have a higher affinity for glucose than for xylose, and the transport inhibition of xylose by glucose can be explained by the similarity between the chemical structures of the two sugars (Sun et al., 2012).

Although many transporter engineering techniques have been applied to solve this issue, some studies that discuss the kinetics of engineered transporters have shown that while their affinity for xylose increases (Km decreases), the transport capacity decreases (Vmax increases) (Gao et al., 2019). In contrast, Young (2012) observed that some mutations can increase transport capacity while decreasing affinity. Consequently, despite the extensive search for specific xylose transporters that guarantee the co-consumption of glucose and xylose, only a few studies have been performed (Reider Apel et al., 2016; Farwick et al., 2014; Nijland and Driessen, 2020; Kuanyshev et al., 2021; Papapetridis et al., 2018).

Elucidation of the XylE transport mechanism has provided sufficient information to relate the xylose transport criteria and their relationship with the structures of their respective transporters. The first crystallography of the partially occluded form of XylE showed that neutral polar amino acids were present at the binding site, and several aromatic amino acids were in the vicinity of the substrate (Sun et al., 2012). The presence of uncharged and nonpolar amino acids in the translocation pore ensures that xylose is transported simultaneously to glucose, whereas naturally occurring XylE has xylose transport activity inhibited by glucose to save energy resources (Sun et al., 2012; Wisedchaisri et al., 2014).

**Figure 4:**
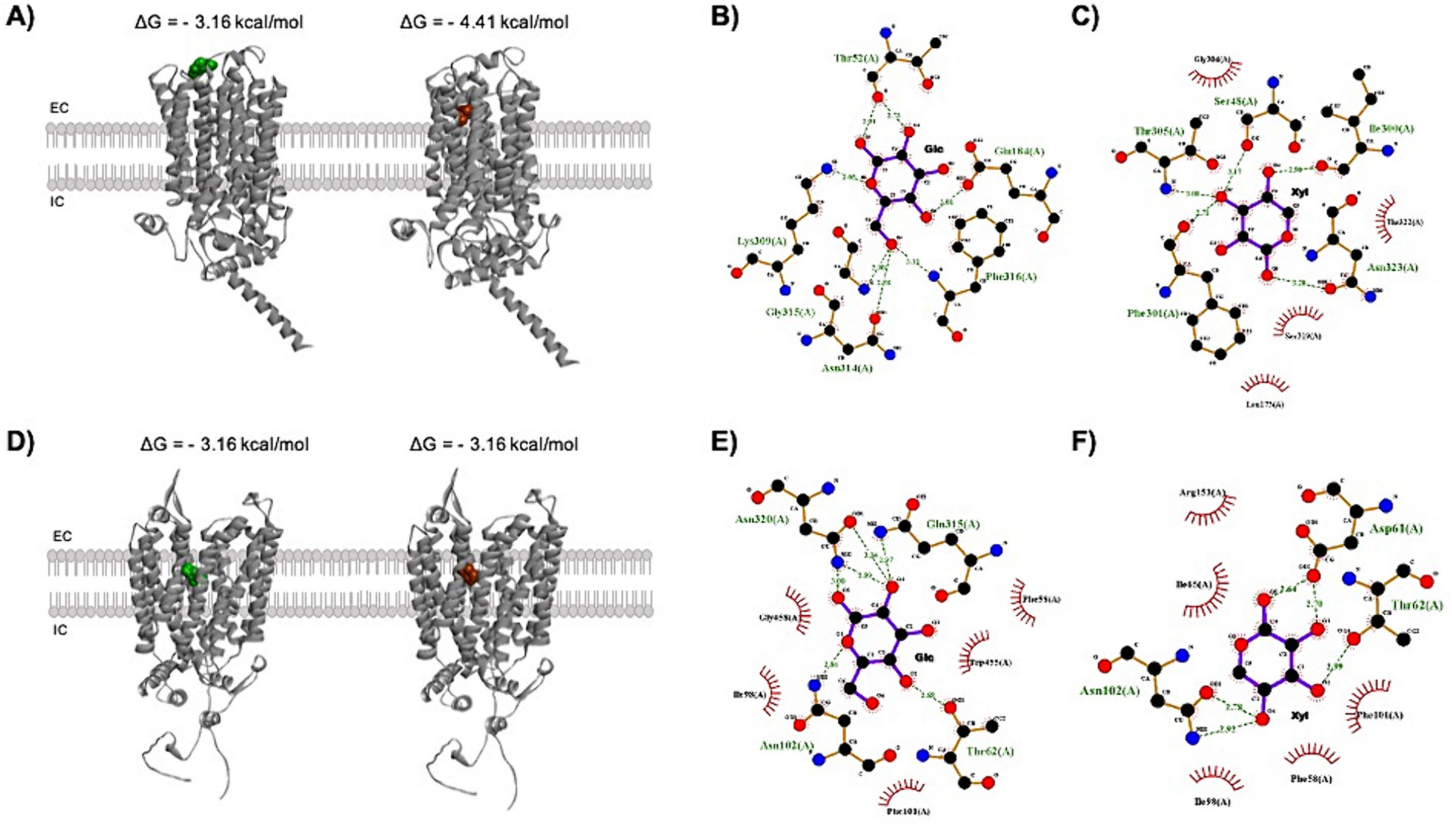
Docking analysis shows different binding affinities for glucose and xylose. **(A)** Tridimensional view of glucose (green) and xylose (red) placements in the Gxs1 transporter outer tunnel with the binding energy values for each of the respective interactions. The interaction between xylose and Gxs1 is clearly more energetically favorable than between glucose and the referred protein. **(B)** The hydrogen bonds (green dashes) and hydrophobic interactions (red semi-circles) formed between glucose and Gxs1, according to the docking analysis. Glucose interacts with some of the residues that form the bottleneck of the tunnel (Stourac et al., 2019), namely Lys309, Asn314, Gly315, and Phe316, in addition to Thr52, which is not part of the bottleneck. **(C)** Xylose also forms hydrogen bonds and hydrophobic interactions with tunnel residues, but different ones: Gly304 and Thr305 are bottleneck residues, whereas Ser48, Ile300, Phe301, and Ser 319 are part of the wider portion of the tunnel. **(D)** 3-D view of the interaction between glucose or xylose and the MFS transporter Gxf1. **(E)** 2D-view of the hydrogen bonds and hydrophobic interactions between Gxf1 and glucose. Glucose interacts with the tunnel-participating residues Thr62, Ile98, and Asn320. **(F)** Xylose interacts with Thr62, Ile65, and Ile98, which are part of the tunnel. Neither glucose nor xylose interacts with bottleneck residues in Gxf1. EC: extracellular; IC: intracellular.

As reported by Leandro et al. (2006), Gxs1 has a higher affinity than Gxf1 (both from *C. intermedia*) for xylose, which may be explained by the number of interactions between Gxs1 and pentose (Figure 4). Gxs1 and Xut3 from *S. stipitis* were used in the first direct evolutionary study of xylose transporters. After several rounds of mutagenesis, we discovered that the mutations were concentrated in three areas of both transporters:(1) at the beginning of the chain, close to TMs 1 and 2; (2) in the middle, next to TMs 6 and 7; and (3) near TMs 11 and 12 (Leandro et al., 2006). Furthermore, for Gxs1, the mutant protein with the highest transport capacity had the most nonpolar substitutions. However, the substitution of E538K in Xut3 and E438K in Gxs1 significantly altered the phenotype and location of the proteins, suggesting that this substitution improves xylose uptake or perhaps changes the general conformation of the protein (Young et al., 2012). Bueno et al. (2020) assessed the likelihood of point mutations changing the affinities of Gxf1, XylE, and Cs4130 for xylose using molecular docking. According to their analyses, mutations that occurred in more distant amino acids (> 5 Å) from the xylose binding site did not influence the affinity of the proteins for the ligand. Furthermore, XylE and Cs4130 share the same xylose binding site, with a high predominance of nonpolar and neutral polar amino acids. This set of features is important for engineering protein targets to optimize the co-consumption of glucose and xylose, and to understand the relationship between the structure and transport of sugars.

*S. cerevisiae* possesses different hexose transporters engineered for xylose fermentation (Sharma and Arora, 2020). Hxt1 is a low-affinity transporter, whereas Gal2 has a high affinity for glucose, which confers higher competitive behavior towards xylose transport through the competitive inhibition of glucose (Hara et al., 2017). Hxt1 is mainly known for its role in the transport of glucose and fructose, although it can also transport xylose despite its lower affinity compared to other xylose transporters, such as Gal2. Studies have revealed that Hxt1 is regulated by various factors, including the concentration of hexoses in the extracellular environment, pH, and the presence of certain metabolites. These regulatory mechanisms help fine-tune the transport of xylose in yeast cells, ensuring that the uptake of xylose occurs in a controlled and efficient manner (Roy et al., 2015).

In Hxt-null *S. cerevisiae* strains that underwent ST site-directed mutagenesis, Gal2 transported xylose without inhibition by glucose, with increased pentose affinity and decreased absorption rates (Farwick et al., 2014). This was the first study to demonstrate the co-utilization of glucose and xylose; structural parallels could be found between this transporter’s structure and that of XylE from *E. coli*, as well as similarities between the mechanisms of the transporters. The previously mentioned N376F mutation in Gal2 almost eliminated the inhibition of xylose transport by glucose and demonstrated that the mechanism of inhibition involves both steric exclusion, which is related to the transporter’s translocation pore size, and competitive inhibition (Farwick et al., 2014). Likewise, the mutation of the chimeric transporter Hxt36, which is composed of the N-terminal portions of Hxt3–438 amino acids and the C-terminal portion of Hxt6–130 amino acids, N367I (N376 in Gal2), also increased the selectivity for xylose, preventing glucose from binding to its site, without affecting the binding of xylose (Nijland et al., 2014; Nijland et al., 2016). The amino acid side chain from the 376th position is oriented toward the sixth carbon (C6) of glucose molecules, and modifications at this site could alter the van der Waals interactions between the surrounding amino acids or even narrow the translocation pore, which could reduce glucose affinity. Both glucose and xylose molecules are complexed in pyranose form; however, only glucose has a C6. Therefore, owing to the differences between xylose and glucose molecules, mutations that lead to amino acid changes at sites that could be related to interactions with the C6 of glucose molecules will probably only affect glucose affinity. As already shown, other studies have performed the same mutation in transporters, replacing this asparagine residue with neutral or nonpolar amino acids, demonstrating that this substitution increases xylose uptake. Consequently, the importance of the presence of aromatic, nonpolar, and neutral, with medium to high-size amino acids in the translocation pore of these proteins is noticeable.

Sequencing analysis revealed a combinatorial set of epistatic mutations at six different positions (L311R, L301R, K310R, N314D, M435T, and T386A) that are crucial for Gal2 improved phenotype. Mutations in residue T386 have been described for Gal2 and other endogenous transporters at homologous positions (Hxt2, Hxt5, and Hxt7). When the T386A mutation was individually tested without combining with other modifications, the strain harboring Gal2 T386A grew in xylose without inhibiting glucose. Curiously, yeast cells harboring the combinatorial Gal2 mutant transporter showed a significantly improved growth profile compared to the single T386A Gal2 transporter. This result showed that the mutation at T386 is related to its affinity for glucose, although mutations in other residues could additionally improve xylose uptake (Reznicek et al., 2015).

Recent studies investigating SWEET transporters have exploited them for yeast engineering, and their ability to co-consume xylose and glucose has been analyzed (Podolsky et al., 2022). Podolsky et al. (2021) found that the wild-type NcSWEET1 transporter from the gut fungus *Neocallimastix californiae* can efficiently transport xylose without glucose inhibition when expressed in *S. cerevisiae* Hxt-null strains. In the mixed culture containing 2.5% glucose and 2.5% xylose, both sugars were consumed almost simultaneously. Based on previous analyses of the resolved structure of the SWEET2b binding site, in *Neocallimastix californiae*, mutant versions of NcSWEET1 were modeled, showing that P52A, and N201A alterations abolished transporter functionality. This effect may highlight the similarity between the proline residues in SWEET and the glycine residues in MFS. In addition to the conserved role of this aromatic residue in binding sugar substrates, it is interesting to note that the mutants P154A and W185G had a decrease of their function. Particularly, about the mutation W185G, its discussed that the maintenance of function is because of the flexibility of the glycine substitution, which enables residue F184 to act as a novel substitute for glucose binding. Emphasis on the significance of the presence of tryptophan residues in the NcSWEET1 binding site can also be pointed out as an important factor for the selectivity of this transporter for xylose.

Another SWEET transporter, LST1_205437, from *Lipomyces starkeyi*, has also been shown to co-consume glucose and xylose (Kuanyshev et al., 2021). In mixed xylose and glucose cultures, the authors compared the transporter with Gal2 from *S. cerevisiae* and observed that LST1_205437 was less inhibited by glucose than Gal2. To understand the differences between the two transporters, the authors analyzed the interactions through molecular docking using structures built based on the outward-facing (OF) and inward-facing (IF) conformations of XylE, and noticed that both transporters have conserved binding sites for xylose and glucose. In the OF conformation, the non-conserved amino acid Y446 of Gal2 forms hydrogen bonds with xylose and glucose. In contrast, in the equivalent position of LST1_205437, there is F433, a nonpolar amino acid that does not form any type of polar bond with the substrate, favoring the transport of xylose. The structure of Gal2 in the IF conformation exposes residue N346, which also forms polar bonds with both substrates. At this position, LST1_205437 contains A335, a nonpolar amino acid residue. Thus, the greater efficiency of LST1_205437 in transporting xylose can be explained by the type of interaction between the transporters. Furthermore, molecular dynamics analyses showed that while LST1_205437 has a competent interaction with the N-and C-terminal domains, xylose does not interact well with the N-terminal domain of Gal2, which may reduce transport efficiency. In addition, a mutation of residue N365 in LST1_205437 (N370/376 in Hxt7/Gal2) was also observed, and the N365V/S/F substitution resulted in increased xylose selectivity.

Engineering of SWEET transporters can play a crucial role in improving the efficiency of bioethanol production from plant-derived sugars. SWEET transporters are responsible for the uptake of sugars into cells, including microorganisms used in bioethanol fermentation. By improving the efficiency of sugar uptake, SWEET transporter engineering can increase the rate and reduce costs associated with bioethanol production. Several studies related to the functional characterization of SWEETs involve the heterologous expression of these transporters in yeasts, where it is possible to verify the improvement in sugar transport using these biotechnological platforms (Xu et al., 2014; Zhang et al., 2021). Recently, Podolsky et al. identified SWEETs in the anaerobic gut fungus, *Neocallimastigomycota*. They expressed fungal NcSWEET1 in transporter-deficient yeast and tested its ability to grow in diverse sugars, including xylose (Podolsky et al., 2021). This ability was due to site-directed mutations that could shift glucose transport to allow xylose to be co-consumed, representing important future perspectives for yeast engineering to optimize biofuel production.

As stated previously, MFS and SWEET transporters share the same alternate access mechanism, the rocker-switch transport cycle, in addition to potentially having a common ancestor, as evidenced by their structural similarities. Therefore, it is anticipated that both types of transporters will be able to transport xylose and glucose together owing to structural factors and the presence of larger hydrophobic amino acids (Xu et al., 2014; Jeckelmann and Erni, 2020; Kuanyshev et al., 2021). Thus, the features that ensure the co-consumption of glucose and xylose in MFS transporters can also be attributed to SWEET transporters.

## 5 Tools for identification of sugar transporters

Increasing the sugar uptake repertoire of microbial cell factories is critical for improving the performance of lignocellulosic hydrolysates (Bueno et al., 2020; Chen et al., 2018; Galazka et al., 2010). However, the number of well-known STs that are beneficial for cell platform engineering approaches remains limited. In addition to identifying STs from fungi, different studies have explored environments that support lignocellulosic breakdown, including the digestive tracts of plague insects (Bueno et al., 2020) and large herbivores, where microorganisms are known to be essential components of the gut microbiota that break down plant biomass into digestible sugars (Podolsky et al., 2021; Seppälä et al., 2016).

**Figure 5:**
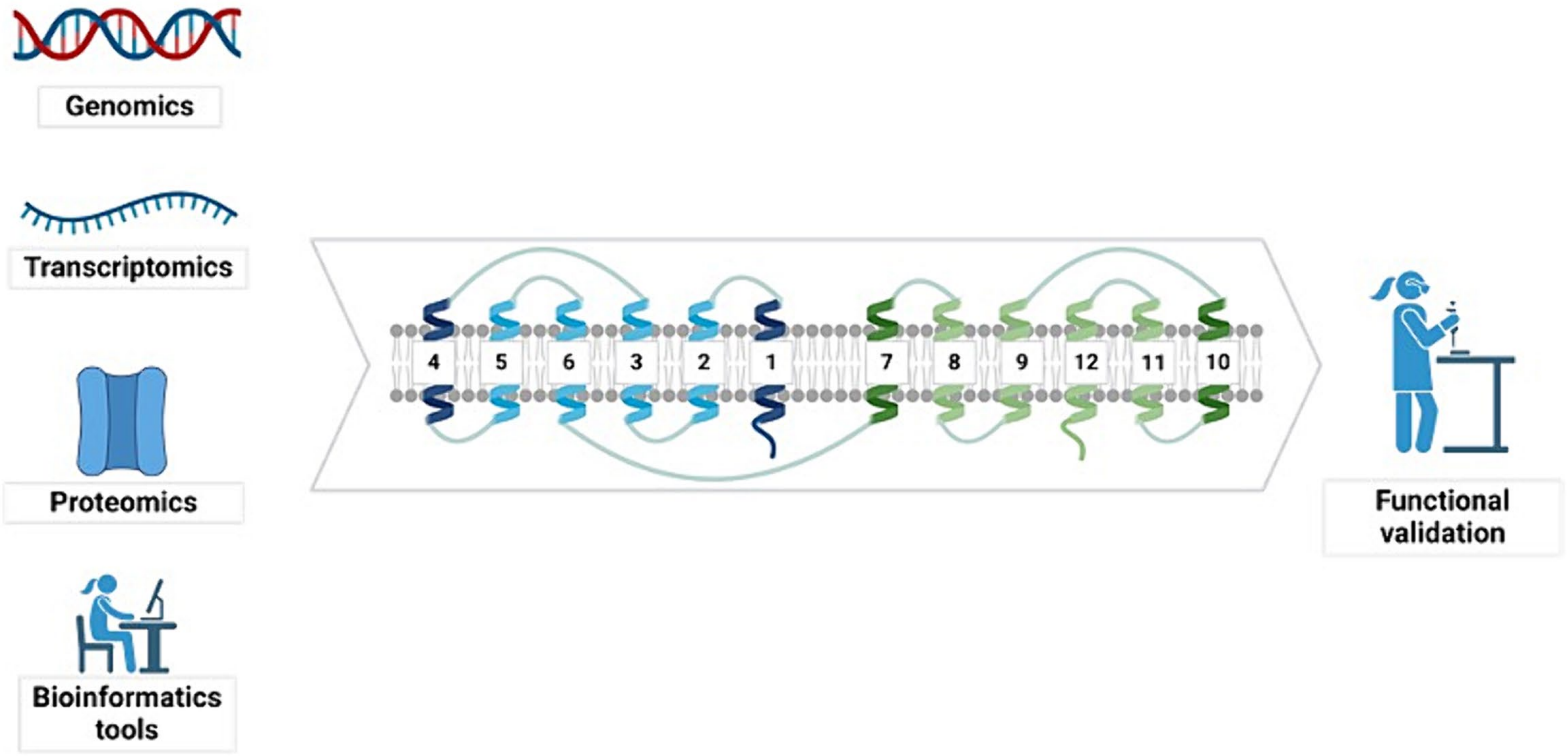
Different tools can be used to identify sugar transporters. Genomics, transcriptomics, proteomics, and bioinformatics tools are applied to identify sugar transporter proteins. The initial identification of sugar transporters must be followed by experimental functional validation. Created with BioRender (Supplementary material S3).

To identify novel sugar transporters of industrial interest, various studies have employed genomic and transcriptomic sequencing, proteomic technologies, and bioinformatics tools (Bueno et al., 2020; dos Reis et al., 2016; Fiamenghi et al., 2022; Sloothaak et al., 2015). These initial steps are essential for providing the information required for the posterior functional characterization of proteins (Figure 5).

Currently, with the vast availability of genomic data, bioinformatics tools enable the prediction of coding sequences and associated protein sequences. Membrane proteins, such as STs, can be predicted from primary sequence data by searching for canonical stretches of 15–22 hydrophobic amino acids that comprise the transmembrane *α*-helical domains. By analyzing the characteristics of the amino acids in the protein, secondary structure prediction methods such as TMHMM (Table 2) can be used to predict the transmembrane segment topology (Huang et al., 2015; Peng et al., 2018). Although membrane proteins can be easily predicted using bioinformatics tools, the set of substrates with which they interact is more difficult to predict. Hidden Markov Models (HMMs), built from a selected list of biochemically characterized transporters, have been described as an effective method to segregate sugar transporters based on their substrates and are also used to scan proteomes for conserved domain structures indicative of conserved functions (Table 2) (Sloothaak et al., 2016; Sloothaak et al., 2015; Finn et al., 2011). Alternatively, functional classification can be performed based on phylogenetic assignment and a collection of homologous, non-redundant transporter sequences identified using a BLAST search (Table 2). However, both approaches have limitations in assessing novel and divergent sequences in newly sequenced microorganisms (Podolsky et al., 2022; Saier et al., 2016).

**Table 2:** Bioinformatics tools applied in the study of xylose transporters.

Tool	Description	Application in the article’s context	Access
BLAST-X	Algorithm to compare a protein sequence with a database of translated nucleotide sequences.	To explore the *T. reesei* genome using cDNA sequences from mono and disaccharide transporters from diverse fungi.	https://blast.ncbi.nlm.nih.gov/Blast.cgi?LINK_LOC=blasthome&PAGE_TYPE=BlastSearch&PROGRAM=blastx
TMHMM	Software for transmembrane protein topology prediction.	To predict the topology and transmembrane helices of transporters.	https://services.healthtech.dtu.dk/services/TMHMM-2.0/
HMM (Hidden Markov Model)	Statistical model that can be used to model biological sequences, such as protein sequences.	To identify xylose transporters by building a specific HMM (HMMxylT) to analyze the proteomes of *A. niger* and *T. reesei*.	http://hmmer.org/
BlastP	Tool for comparing a protein sequence against a database of protein sequences.	To identify orthologs of known sugar transporters in the genome of *Lipomyces starkeyi*, searching for conserved motifs such as GG/FXXXG, T213, and N370.	https://blast.ncbi.nlm.nih.gov/Blast.cgi?PAGE=Proteins
Machine Learning Algorithms	Use statistical methods to find patterns in datasets.	Construction of a classification model to identify xylose transporters in yeasts.	Generic methods.
AlphaFold	Artificial intelligence system that predicts the three-dimensional structure of proteins.	Modeling the structure of the NcSWEET1 protein from *Neocallimastigomycota*.	https://colab.research.google.com/github/sokrypton/ColabFold/blob/main/AlphaFold2.ipynb#scrollTo=kOblAo-xetgx
BioEdit	Multiple sequence alignment tools.	To compare amino acid sequences of different xylose transporters (e.g., XylE, Str1, Str3, Mgt05196p, Hxt7, Gxs1, and Gxf1).	https://thalljiscience.github.io/
Jalview	Software to visualize and analyze sequence alignments.	Visualization of the multiple sequence alignment of proteins.	https://www.jalview.org/
HMMtop	Web server for transmembrane protein topology prediction.	Prediction of the topology and transmembrane helices of proteins such as XylE, Str1, Str3, Mgt05196p, Hxt7, Gxs1, and Gxf1.	http://www.enzim.hu/hmmtop/
TMRPres2D	Software to generate high-quality visual representations of transmembrane protein models.	Generation of images of the topology of proteins such as XylE, Str1, Str3, Mgt05196p, Hxt7, Gxs1, and Gxf1.	http://bioinformatics.biol.uoa.gr/TMRPres2D/

Several studies have exploited transcriptomic and proteomic tools for gene mining and functional identification. Lignocellulolytic microorganisms possess a sophisticated regulatory circuit that efficiently controls the expression of a set of proteins, including sugar transporters, that are specific for the utilization of different sugars (Akel et al., 2009; Dos Santos et al., 2016; dos Santos et al., 2014; Ries et al., 2016). Taking advantage of this, the analysis of the global transcriptome and proteomic responses of these microorganisms to different carbon source conditions offers insights into the specific sugar transporters required for the utilization of a particular carbon source (dos Santos et al., 2014; Bischof et al., 2013). Generally, these studies also involved the analysis of wild-type and ST knockout strain cells in discriminating carbon sources to understand sugar transporter functions.

To identify STs, proteomic analysis has also been reported for proteins isolated from the cell membranes of microorganisms grown under specific carbon source conditions (Sloothaak et al., 2015). A plasma membrane proteome under sugar-specific conditions can provide a more precise source of information to identify the most critical sugar transport components involved in the sugar uptake process. This is because, in addition to providing a valuable alternative to genome-wide sequencing for gene mining and functional identification, transcriptome sequencing does not account for regulatory events at the post-transcriptional level, even though these events can impact protein abundance and localization. To answer these specific questions, both transcriptomic and proteomic data must be processed using bioinformatics. These approaches are especially useful when searching for distantly related transporters such as those from different families (Sloothaak et al., 2016; Sloothaak et al., 2015).

Initial identification of sugar transporters must be followed by functional validation. The most commonly applied methods for sugar transporter functional validation include an *in vivo* analysis by deleting the sugar transporter gene in the endogenous host and an *ex vivo* analysis in a host without a specific transporter system, natively or developed by genetic engineering approaches, and harboring a specific consumption pathway. In the first method, the inefficiency of function in the endogenous host enables the investigation of the resulting phenotype, including quantification of the substrate uptake rate (Huang et al., 2015; Nogueira et al., 2018). In the second method, microorganism growth on a specific metabolizing sugar was used to confirm the putative sugar transporter function, and transport assays using radiolabeled sugars were used (Bueno et al., 2020; dos Reis et al., 2016; Dos Reis et al., 2013).

Recently, electrophysiological methods have been described for investigating the STs. Using this approach, the selected transporters can be expressed in *Xenopus laevis* oocytes and screened for transport activities. A complete set of kinetics can be simultaneously determined for a single oocyte at several test voltages at the same time. Although electrophysiological investigations are useful for transporter functional characterization because they do not require expensive and scarce radiolabeled sugars, this technique is limited to the characterization of electrogenic transporters such as sugar/H+ symporters (Havukainen et al., 2021).

Taking advantage of these strategies, diverse studies have described a combination of approaches for the identification and functional characterization of xylose transporters. Huang et al. (2015) identified a sugar transporter essential for the utilization of pentoses in *T. reesei*. The BLAST-X algorithm was applied to explore the *T. reesei* genome using mono and disaccharide transporter cDNA sequences from diverse fungi available in the NCBI GenBank (Table 2). Five amino acid sequences belonging to the major facilitator superfamily, including TrStr1, were identified. Using TMHMM software, the TrStr1 topology was predicted, also showing conserved key residues that have been described to be involved in either sugar specificity or substrate binding in some structure-solved sugar transporters (Table 2). Phylogenetic analysis of TrStr1 and its homologs indicated that TrStr1 is a putative monosaccharide transporter. Finally, TrStr1 expression in an engineered *S. cerevisiae* hxt-null strain and gene deletion in *T. reesei* enabled functional validation of this transporter, showing its involvement in pentose uptake.

Sloothaak et al. (2016) identified xylose transporters using a different strategy: a hidden Markov model (HMMxylT) developed specifically for xylose transporters to explore and filter the *in silico* proteomes of *A. niger* and *T. reesei*. Considering the characteristics of transporters expressed by filamentous fungi that can often transport more than one type of sugar, it has been reported that HMMs may be used to segregate sugar porter proteins based on their substrates more successfully than typical BLAST-based approaches. However, precision depends on the availability of a consistent training set of previously described proteins with the functions of interest (Sloothaak et al., 2016).

To construct this model, protein sequences from functionally confirmed xylose transporters were chosen with the additional condition that they originate from microorganisms that naturally metabolize xylose. The authors compared the HMMxylT output with transcriptome analysis studies from *A. niger* cultures in the presence of diverse carbon sources, including xylose and sugarcane bagasse. Phylogenetic analysis was also performed on the selected top-scoring candidate transporters, considering the transporters described in the literature. Using this approach, a list of five putative xylose transporters were obtained: three from *A. niger* (XltA, XltB, and XltC) and two from *T. reesei* (Str2 and Str3). A previously described Str1 strain was also identified in this study. Putative transporters were functionally validated in an *S. cerevisiae* HXT-null strain harboring a xylose consumption pathway. However, all sugar transporters transported xylose with varying affinities (Sloothaak et al., 2016).

Recently, a study has shown a bioprospecting approach was developed for the discovery of novel structurally distant sugar transporters from previously unexplored oleaginous yeasts and plants that are capable of co-consuming glucose and xylose. The authors identified a transporter from *Arabidopsis thaliana* that can transport glucose and xylose simultaneously without inhibition. By screening 17 AtSWEET1-17 transporters belonging to the SWEET family, AtSWEET7 was found to confer glucose and xylose co-consumption abilities in the engineered *S. cerevisiae* SR8D8 strain (Kuanyshev et al., 2021). Conversely, to identify orthologs of known sugar transporters in the genome sequence of *Lipomyces starkeyi*, a BlastP tool was applied using the search criteria based on the conserved motifs GG/FXXXG, T213, and N370 residues, signatures described to be involved in Hxt7 xylose specificity. Among the transporters obtained, LST1-76 was identified as a putative xylose transporter. Further expression in engineered *S. cerevisiae* lacking major hexose transporters (SR8D8) revealed the intrinsic ability of this protein to co-consume glucose and xylose (Kuanyshev et al., 2021).

Furthermore, Fiamenghi et al. (2022) described the application of machine learning and comparative genomic approaches for identifying xylose transporters. The authors obtained a classification model that enabled the selection of four sugar transporter candidates by implementing a strategy that searches for motifs that may be involved in past adaptations and are responsible for xylose transport in xylose-fermenting species. Posterior functional characterization of *S. cerevisiae hxt*-null strain confirmed the xylose uptake function of the transporter (Fiamenghi et al., 2022). Another tool used to identify sugar transporters is Machine Learning, which uses statistical methods and algorithms to explain and find patterns in datasets that would be extremely difficult through conventional means and experiments. Over the last decade, these approaches have gained traction in different biological investigations. A few examples include the prediction of sequence specificity in nucleic acid-binding proteins (Alipanahi et al., 2015), differentiation between mRNA and lncRNA sequences (Camargo et al., 2020), metabolic system evolution studies (Konno and Iwasaki, 2023), and the more famous Alphafold2 algorithm (Jumper et al., 2021), which promises to solve all protein structures without the need for crystallographic data. Several reviews have addressed the general uses of machine learning in biology in greater depth (Ching et al., 2018; Greener et al., 2022; Libbrecht and Noble, 2015).

Considering the diverse studies showing the relevance of an efficient transport system suitable for developing *S. cerevisiae* towards industrial applications, active research on sugar transporter identification, combined with protein engineering efforts, will boost the activity of existing transporters and potentially overcome transport flux bottlenecks in engineered strains.

Comparative genomics and evolutionary studies can also be used to shed light on the functional, structural, and adaptive traits of sugar transporter proteins as well as to help classify and group transporters of similar origin and descent, as seen with the MFS and SWEET STs. As stated previously, MFS transporters are highly structurally conserved and are believed to have diverged from initial gene duplication before their separation into subfamilies. Sugar transporters in the MFS are found mainly in the Sugar Porter, Oligosaccharide:H+ symporter, and Fucose-galactose-glucose:H+ symporter subfamilies, with sugar porters having the most members (Pao et al., 1998). A correlation between the substrate carried and the phylogenetic history of a transporter family is seen (Palma et al., 2007; Gonçalves et al., 2016), but Sugar Porters have a large degree of non-specificity in transported sugars, rendering it difficult to predict sugar specificity and transport capacity through phylogenetic history alone (Lazar et al., 2017). As seen in the third topic of this review (Young et al., 2014) MFS sugar transporters are usually under purifying selection (Wang et al., 2016), meaning that changes in amino acids are usually deleterious or costly for organisms; therefore, an alternative evolutionary strategy for increasing sugar transport capacity has been reported, based on gene expansion and duplication of transporter families, such as the Hxt transporters in Crabtree-positive yeasts (de Valk et al., 2022; Lin and Li, 2011) and the KHT and HGT families in *Kluyveromyces marxianus*. Dose dependency and gene duplication facilitate sugar uptake in sugar-rich environments and help against competition in stressful conditions with low sugar concentrations (Kondrashov, 2012; Brown et al., 1998), at the energetic cost of maintaining additional copies in the genome (Wagner, 2005; Adler et al., 2014). Gene duplication, although encumbered by fitness costs and instability, which reduce the likelihood of both sub-and neofunctionalization, may allow positive selection for novel beneficial functions (Adler et al., 2014), allowing one duplicated copy to gain affinity more easily for an alternative sugar than originally transported.

SWEETs are members of the transporter/opsin/G protein-coupled receptor (TOG) superfamily, which is believed to be a 2TM protein that gives rise to a 4TM protein and all other members of this superfamily (Yee et al., 2013). A wide phylogenetic analysis of SWEETs has shown segregation among plants, bacteria, and animals, with fungi, algae, and *Oomycota* clustering. In bacteria, SWEETs have extensively diverged into multiple groups, although a shared common ancestor can be traced (Jia et al., 2017), and there is little information about fungal SWEETs. Although phylogenetic history is associated with some individual traits, it is generally insufficient to explain the associations between traits (Opulente et al., 2018). Nevertheless, comparative genomics can be a powerful method for prospecting genes of industrial interest, and more recently, it has been used to choose transporter candidates for industrial applications, such as second-generation ethanol production (Wohlbach et al., 2011; Riley et al., 2016; Lu et al., 2021). This strategy has become more prevalent owing to the increasing availability of whole-genome sequencing data for different species (Lu et al., 2021; Shen et al., 2018; Peter et al., 2018), resulting in a wide variety of genes in other organisms that may be better adapted to the consumption of sugars, such as pentoses, than those currently studied (Riley et al., 2016; Shen et al., 2018). This approach involves understanding the evolutionary history of these organisms and identifying evolutionary clues and markers that suggest that a sugar transporter is better suited for industrial use. Interesting candidates for wet laboratory experimental validation have been suggested using this screening method (Bueno et al., 2020; Fiamenghi et al., 2022). Further characterization and comparison studies are essential to discover and unlock the full potential of sugar transporter candidates for use in industrial yeasts, including studies with other less-explored superfamilies, such as SWEETs, as they can bring novel traits and strategies for the optimization of current strains.

Studies on transporter proteins are still incipient, and much of the work has been done on classifier algorithms aiming to either segregate membrane/transporters from non-membrane proteins (Alballa and Butler, 2020), identify and separate them into different transporter classes according to sequence features (Lin et al., 2006), predict overall substrate specificity (Mishra et al., 2014), or identify proteins of unknown function as ABC transporters (Hou et al., 2020). Research on sugar transporters is emerging, aiming to classify glucose transporters into their respective superfamilies, for instance (Ali Shah et al., 2021) or predict xylose transport capacity (Fiamenghi et al., 2022), the latter showing promising results to serve as an initial screening strategy for finding novel xylose transporters. Another recent study created a model to predict structural conformations in different states from the Sugar Porter family, trying to understand the molecular and evolutionary clues of the transport mechanism (Mitrovic et al., 2022), which in turn could also be useful for predicting the specificity of sugar transporters or how certain transporters behave when in contact with C5 against C6 sugars.

Some studies have shown that evolutionary features, such as the position-specific scoring matrix (PSSM), have significant importance in model output (Lian et al., 2018), and it has also been shown, albeit with other interests, that it is possible to study comparative genomics through machine learning models (Iwasaki et al., 2022), highlighting the possibility of combining comparative genomics and machine learning methodologies (Table 2).

## 6 Strategies to engineer xylose transporters for improved assimilation

Adaptive Laboratory Evolution (ALE) is an efficient methodology widely used to improve different phenotypes of microorganisms for biotechnological applications (Table 1). In ALE studies, a defined condition is set during a prolonged cultivation period, and natural mutations arise over time and increase in the cell population. Under the selective pressure of evolution, beneficial mutations are fixed to the population by natural selection and increase in frequency if selective pressure continues to be imposed under the established experimental conditions. After many generations, cells with improved fitness accumulate a set of specific mutations that are associated with an improved phenotype of interest.

ALE studies have focused on modifying yeast strains to improve xylose consumption. Many studies using ALE to generate cells with high xylose consumption have found mutations reported in the literature are the first to identify genes that affect xylose metabolism (Wisselink et al., 2009; Verhoeven et al., 2018). One strategy for enhancing the selective pressure on sugar transporters is to use a specific yeast platform capable of imposing this evolutionary pressure on the transport system, such as a hexose transporter null strain, *hxt*-null. This strain has the main endogenous hexose transporter deleted from its genome, which prevents the cells from growing in glucose and xylose. The cellular growth profile can be restored by expressing a specific sugar transporter. Therefore, in an ALE experiment, selective sugar uptake pressure is exerted solely on this transporter, although other mutations may occur throughout the genome. Moreover, coupled with the deletion of the main hexose transporters, disruption of the glucose pathway by multiple hexokinase knockouts (*hxk*-null), which prevent glucose from being utilized as a carbon source, is a powerful combination to impose selective pressure on the transport system for C6/C5 co-fermentation. The first study using the ALE approach to improve xylose transport with an *hxt-hxk*-null strain expressing the xylose consumption pathway was reported by Farwick et al. (2014). Different mutants capable of growing on xylose in the presence of glucose were selected for an ALE experiment with *hxt/hxk*-null yeast cells expressing endogenous Gal2, Hxt7, and Hxt5 transporters (Farwick et al., 2014).

Different ALE experimental sets have been used in other studies; for example, using only a hexokinase-null strain capable of efficiently consuming xylose. However, Nijland et al. (2014) selected an evolved strain with all endogenous sugar transporters present in its genome, capable of consuming xylose without glucose inhibition in their ALE experiments. Genome sequencing of the best-evolved strain indicated the fusion of the endogenous transporters Hxt3 and Hxt6, creating the chimeric transporter Hxt36. A similar experimental design was reported by the same research group. An ALE experiment was performed in a xylose-fermenting yeast strain with deletions in hexokinase genes (*hxk1, hxk2, glk1,* and *gal1*). However, in contrast to previous reports, genomic sequencing of the best-evolved isolate revealed a mutation in the general transcriptional corepressor Cyc8 (Y353C) (Reznicek et al., 2015). Further investigation of this SNP indicated that genes from the *hxt* family had their expression increased, particularly *hxt1, hxt2, hxt36, hxt5*, and *hxt7*. Therefore, this point mutation in Cyc8 was indirectly responsible for changing the transporter landscape, without any genetic alteration to the transporter protein structure, by increasing the availability of these endogenous transporters and favoring the uptake of xylose and glucose. Other studies have reported various mutations related to improved sugar uptake in different strains and experimental sets for ALE. Therefore, from this variety of ALE experiments, it is possible to identify different modified sugar transporters with specific mutations that can enhance xylose affinity and abolish glucose inhibition for specific xylose uptake.

Alternatively, in the ALE approach, where mutations arise naturally and can be selected by evolutionary pressure over generations, direct evolution relies on random mutagenesis approaches that increase the mutation rate in specific regions coupled with the rapid screening of mutants with improved phenotypes. The development of mutant transporter libraries for direct evolutionary approaches is most commonly achieved using PCR-based methodologies (Table 1). Traditional error-prone PCR (epPCR) is based on low DNA polymerase fidelity under predetermined conditions (MgCl2 concentration and alternative polymerase cofactors) to increase the mutation rate during PCR cycles. However, any mutation can appear in the coding region of the target gene, including mutations that could induce a premature stop codon or even base indel (insertions and/or deletion), inactivating the target protein. Therefore, using this error-prone approach for high-throughput screening and a suitable host-expressing organism, it is possible to cover the largest possible share of positive mutants in this protein library.

Different approaches for building synthetic protein libraries have emerged in the last decade. An alternative method to reduce these errors is site-directed mutagenesis (Table 1). However, when there is limited information about the available proteins, such as reported positive mutations or reliable crystallographic/modeled structures, this alternative is not very effective. Therefore, methodologies that use broad random mutagenesis of the entire DNA sequence of the target gene are indicated for this particular case. SpeedyGenes and its current version, SpeedyGenesXL, are modern methodologies that include error correction procedures during the synthesis of protein libraries. Using endonuclease treatment, which recognizes and cleaves possible errors (insertions, deletions, or mismatches), it is possible to create a protein library of large genes (>1,500 bp) that exhibit positive activity. Depending on the target protein and information available in the literature, different methodologies can be applied to create and screen mutant synthetic protein libraries.

Young et al. (2012) reported the first direct evolutionary study of sugar transporters for biotechnological purposes. In this study, an epPCR approach in two heterologous transporters, Gxs1 and Xut3 from *C. intermedia* and *S. stipitis*, respectively, coupled with library screening, revealed several amino acid residues that are important for transport activities, as discussed in the previous sections. Since this first report using the direct evolution of sugar transporters, various other studies using mutagenesis techniques have been published (Lian et al., 2018; Reznicek et al., 2015; Wang et al., 2016). Eight different mutants with improved xylose transport were selected from the endogenous Gal2 transporter using three rounds of error-prone PCR cycles, coupled with high-throughput screening by flow cytometry (Reznicek et al., 2015). Therefore, random direct evolution strategies are powerful and simple approaches that allow the exploration and discovery of novel residues that cannot be rationally predicted. Coupled with a large screening system, this approach allows the identification of promising mutations in sugar transporters that can significantly improve xylose uptake rates and provides valuable information regarding the importance of each residue in sugar transport and its combinatorial epistatic effect to enhance xylose assimilation.

Both direct and adaptive laboratory evolution approaches (Table 1), along with structural data and crystallographic or modeling structures, can provide essential information on the molecular mechanisms that regulate sugar uptake dynamics. Based on these methodologies, important amino acid positions have been suggested to be related to xylose specificity. As mentioned in the ALE experiment, position N376 in the Gal2 transporter is responsible for abolishing glucose uptake (Farwick et al., 2014). Moreover, using different experimental sets, amino acid positions homologous to N376 described in Gal2 were reported for a different sugar transporter, Hxt36 (Nijland et al., 2014). As discussed in previous studies, the specific positions discovered by ALE experiments, coupled with the modeled structures of each transporter, are related to the intermolecular interactions between sugar molecules and the side chain of amino acids in the protein. Therefore, from this and other available information regarding amino acid positions and their interactions in sugar transport, it is possible to rationally modify sugar transporters by site-directed mutagenesis.

Using a rational approach (Table 1), both endogenous and heterologous mutant sugar transporters with improved xylose uptake have been reported (Jiang et al., 2020; Farwick et al., 2014; Nijland and Driessen, 2020; Wang M. et al., 2015). Wang C. et al. (2015) and Wang M. et al. (2015) extensively analyzed putative sugar transporters from the xylose-consuming yeast *Meyerozyma guilliermondii* and determined that the Mgt05196p transporter was the best xylose transporter from this host organism. Moreover, comparing the crystal structure from the *E. coli* XylE sugar transporter with the *in silico* structural model of Mgt05196p, 28 amino acids were targeted to site-directed mutagenesis.

In the work of Young et al. (2014), similarly to that of Farwick et al. (2014), the ALE experiment in the *hxt-hxk*-null strain allowed the acquisition of sufficient information to understand the best amino acid change at this site and, consequently, to develop the best xylose transporter variants (Farwick et al., 2014). Thus, rational design by site-directed mutagenesis might be a powerful approach to modify sugar transporters to increase desirable features, such as xylose uptake rates and specificity. However, previous information regarding transport dynamics, such as beneficial mutations that highlight possible hotspots, must be known for this methodology to be used.

## 7 Conclusion

As is already well established in structural biochemistry, the amino acid sequence of a protein provides a lot of information about its structure and function. Xylose transporters, as membrane proteins, have a well-conserved three-dimensional structure. Therefore, although the similarity between their primary sequences is quite low, these proteins have very well conserved motifs and amino acids that are fundamental for maintaining their activity and structure. These motifs and amino acids also function as modification targets to optimize xylose transport for various purposes, such as second-generation ethanol production. Therefore, this work compiled knowledge about the structural characteristics of xylose transporters and their transport roles based on structure–function relationships. Furthermore, in this study, we compiled several tools for identifying xylose transporters, from computational techniques, bioinformatics, to experimental techniques. Furthermore, this work brings together approaches for engineering these transporters to optimize xylose transport, increasing the specificity of native xylose transporters for the pentose or by the improvement of transport capacity.
