# Supplementary material for: Structural and biochemical insights of xylose MFS and SWEET transporters in microbial cell factories: challenges to lignocellulosic hydrolysates fermentation
Source: Front Microbiol. 2024 Sep 27;15:1452240. doi: 10.3389/fmicb.2024.1452240 (PMC11466781; doi:10.3389/fmicb.2024.1452240)
Supplement: Supplementary file 1 [file Image_1.pdf]

49 Spadina Ave. Suite 200
Toronto ON M5V 2J1 Canada
www.biorender.com

Confirmation of Publication and Licensing Rights

August 22nd, 2024
Science Suite Inc.

Subscription:	*Student Individual - Academic*
Agreement number:	*UC277QRYQ5*
Publication name:	*Frontiers in Microbiology*
Citation to Use:	*Created with BioRender.com*

To whom this may concern,

This document is to confirm that Iasmin Taveira has been granted a license to use the BioRender Content, including icons, templates, and other original artwork, appearing in the attached Completed Graphic pursuant to BioRender's Academic License Terms. This license permits BioRender Content to be sublicensed for use in publications (journals, textbooks, websites, etc.).

All rights and ownership of BioRender Content are reserved by BioRender. All Completed Graphics must be accompanied by the following citation: "Created with BioRender.com".

BioRender Content included in the Completed Graphic is not licensed for any commercial uses beyond use in a publication. For any commercial use of this figure, users may, if allowed, recreate it in BioRender under an Industry BioRender Plan.

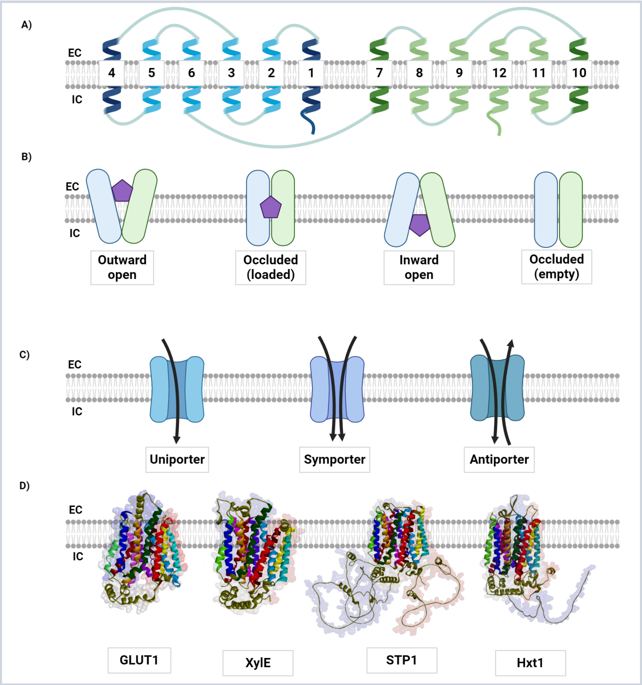

For any questions regarding this document, or other questions about publishing with BioRender refer to our BioRender Publication Guide, or contact BioRender Support at support@biorender.com.
